# A modified liquid chromatography/tandem mass spectrometry method for predominant disaccharide units of urinary glycosaminoglycans in patients with mucopolysaccharidoses

**DOI:** 10.1186/s13023-014-0135-3

**Published:** 2014-09-02

**Authors:** Chih-Kuang Chuang, Hsiang-Yu Lin, Tuen-Jen Wang, Chia-Chen Tsai, Hsuan-Liang Liu, Shuan-Pei Lin

**Affiliations:** Division of Genetics and Metabolism, Department of Medical Research, Mackay Memorial Hospital, Taipei, Taiwan; Department of Pediatrics, Mackay Memorial Hospital, Taipei, Taiwan; Department of Laboratory Medicine, Mackay Memorial Hospital, Taipei, Taiwan; Institute of Biotechnology, National Taipei University of Technology, Taipei, Taiwan; College of Medicine, Fu-Jen Catholic University, Taipei, Taiwan; Department of Early Childhood Care and Education, Mackay Junior College of Medicine, Nursing and Management, Taipei, Taiwan; Department of Medicine, Mackay Medical College, New Taipei City, Taiwan; Institute of Clinical Medicine, National Yang-Ming University, Taipei, Taiwan; Department of Infant and Child Care, National Taipei University of Nursing and Health Sciences, Taipei, Taiwan

**Keywords:** Enzyme replacement therapy, Glycosaminoglycans, Liquid chromatography/tandem mass spectrometry, Methanolysis, Mucopolysaccharidosis

## Abstract

**Background:**

The identification of acid mucopolysaccharide by the liquid chromatography/tandem mass spectrometry method (LC-MS/MS) of the predominant disaccharide units of glycosaminoglycans (GAGs) (chondroitin sulfate, CS; dermatan sulfate, DS; heparan sulfate, HS) after methanolysis is validated and applicable for mucopolysaccharidosis (MPS) type determination.

**Methods:**

A total of 76 urine samples were collected and analyzed, from nine MPS I patients, 13 MPS II patients, seven MPS III patients, eight MPS VI patients, and 39 normal controls. Urinary GAG was first precipitated by the Alcian blue method followed by a treatment of 3 N HCl methanol. The protonated species of the methylated disaccharide products were detected by using a multiple reaction monitoring experiment. Internal standards, [^2^H_6_] CS, [^2^H_6_] DS and [^2^H_6_] HS, were prepared in-house by deuteriomethanolysis of CS, DS and HS.

**Results:**

One particular disaccharide for each GAG was selected, in which the parent ion and its daughter ion after collision were *m/z* 426.1 → 236.2 for DS (*m/z* 432 → 239 for dimers derived from [^2^H_6_] CS and [^2^H_6_] DS) and *m/z* 384.2 → 161.9 for HS (*m/z* 390.4 → 162.5 for the [^2^H_6_] HS dimer). The quantities of DS and HS were determined, which varied from one MPS type to the other. The results can be used to evaluate the severity of MPS subgroups, as well as urinary GAG amelioration at follow-up after enzyme replacement therapy (ERT).

**Conclusions:**

The modified LC–MS/MS method for MPS type determination is specific, sensitive, validated, accurate, and applicable for simultaneous quantifications of urinary DS and HS. This method can help to make correct diagnosis of MPS patients and evaluate the effectiveness of ERT.

## Introduction

The mucopolysaccharidoses (MPS), known as a group of lysosomal storage disorders, are caused by the deficiency of specific enzymes that catalyze the stepwise degradation of glycosaminoglycans (GAGs; mucopolysaccharides). There are at least 11 known enzymes involved in the catabolism of dermatan sulfate (DS), heparan sulfate (HS), keratan sulfate (KS), chondroitin sulfate (CS), and hyaluronic acid (HA) [1–3]. Any enzyme deficiency will block the GAG degradation, which results in GAG macromolecules with a specific carbohydrate or sulfated carbohydrate residue accumulating in cells, causing severe dysfunction [1–4]. The clinical manifestations of MPS are chronic and progressive. The severity and prognosis vary from one type to another with a wide spectrum of clinical severity. MPS patients present with many distinct clinical features, including organomegaly, developmental delay, dysmorphic facial features, skeletal dysplasia (dysostosis multiplex), and growth retardation. In addition, hearing [5], vision (mostly with corneal clouding), pulmonary and cardiovascular function [6,7], bone mineral density [8], and joint mobility are also affected. The MPS clinical features may present from birth to late childhood or even in early adulthood, depending on the severity of the MPS type. Death from respiratory or cardiac failure and respiratory infections usually occurs before the age of 10 years in severe phenotypes [1–4,9].

An accurate diagnosis is achieved generally by three sequential tests: the quantification of urinary GAG, two-dimensional electrophoresis (2-D EP) qualitative analysis, and leukocyte enzyme activity assay [10–12]. Urinary GAG quantitative analysis can provide a screen for MPS diagnosis, but cannot be used to identify a specific MPS disease. A 2-D EP is the most common and feasible method used for specific MPS disease. Nevertheless, this method is time-consuming and requires more than two days to complete a test. Moreover, the interpretation is difficult and subjective, which can make the diagnosis unreliable. Particularly, the interpretation of the 2-D EP pattern is ambiguous due to the affected GAGs being similar in MPS I, II, and VI. Based on the limitations of MPS first-line screening methods, the establishment of an advanced liquid chromatography/tandem mass spectrometry method (LC-MS/MS) for MPS subgroup determination is valuable and important.

The polysaccharide chains are elongated by the sequential addition of alternating acidic and amino sugars, donated by their UDP-derivatives. Amino sugars are essential components of GAGs, glycoproteins, and glycolipids. Members of the GAG family vary in the type of hexosamine, hexose or hexuronic acid unit they contain (e.g. glucuronic acid, iduronic acid, galactose, galactosamine, glucosamine) [13]. The specific GAGs of physiological significance are DS, CS, HS, KS, and HA. Although each of these GAGs has a predominant disaccharide component, heterogeneity does exist in the sugars presented in the make-up of any given class of GAG. In this study, the identification of acid MPS by the modified LC-MS/MS method of the predominant disaccharide component of GAGs after methanolysis [14–18] is proposed and validated for MPS type determination [19,20].

## Materials and methods

The LC-MS/MS method for GAG disaccharide analysis was modified according to the literature reported by Auray-Blais et al. [19]. For GAG disaccharide LC-MS/MS analysis, a multiple reaction monitoring (MRM) experiment giving a precursor molecular ion (Q1) and a respective fragment ion (Q3) was normally applied. The instrument was operated in a positive-ion ([M + H]^+^) mode. The quadrupoles, Q1 and Q3, were tuned with unit resolution, and the MS conditions were optimized for maximum signal intensity. The LC-MS/MS analysis was performed on a 4000 QTRAP LC-MS/MS system (AB Sciex, Foster City, CA, USA) equipped with a TurboIonSpray (electrospray ionization; ESI), and Agilent 1260 Infinity HPLC pump and autosampler (Agilent Technologies, Santa Clara, CA, USA). The experimental parameters were set and are clearly described in the following section. Data were acquired and processed using Analyst 1.5.2™ software (AB Sciex). Calibrations of GAG standards and internal standards were performed with every batch of samples.

### MPS patient details and normal control

A total of 76 urine samples were collected and analyzed from nine MPS I patients, 13 MPS II patients, seven MPS III patients, eight MPS VI patients, and 39 normal controls. MPS I phenotypes comprised five MPS I-Hurler/ Scheie (H/S) and four MPS I-S patients according to their progressive central nervous system and the severity of somatic manifestations. They have received enzyme replace therapy (ERT) in a varied duration, from one month to 8.8 years, and the ages of ERT initiation were from 0.7 to 34.9 years old. In those, two patients expired due to respiratory infection and complications. For MPS II phenotypes, eight severe and five mild forms were inclusive in this study. All 13 patients have received ERT and the duration of receiving ERT was from 1.3 to 7.3 years. The ages of ERT initiation were varied, from 4.5 to 18.3 years old. The seven MPS III patients were identified as one IIIA, three IIIB, and three IIIC and all patients were confirmed by leukocyte enzymatic assays. No MPS VI phenotypes were further classified. Soft tissue and skeletal disease presented in MPS VI patients, but without central nervous system (CNS) involvement. These patients have received ERT from 3 to 10.3 years, and the ages of ERT initiation were from 4.4 to 21.2 years old. Normal controls (n = 39) were chosen from out-patient department, and informed consent was obtained from all normal population and MPS patients. The study was approved by the ethics committee of Mackay Memorial Hospital, Taipei, Taiwan.

### GAG precipitation and methanolysis

Urine samples (approximate 10 mL) were collected in a sterile urine container (polyethylene; Nalge Nunc International, USA). The method of GAG precipitation was described previously [10–12]. GAGs were first precipitated from urine using the Alcian blue (AB) reagent containing sodium acetate. Then, sodium chloride and methanol were used to dissolve MPS-AB complex. Sequentially, sodium carbonate was added to dissociate the MPS complex and AB. Finally, ethanol was used to reprecipitate MPS. After evaporated to dryness, the precipitate was dissolved in water based on dimethylmethylene blue value in mg/L, and the supernatant was subjected to methanolysis based on the method reported by Chambers RE et al. [21]. The GAGs were then degraded to uronic acid-*N*-acetylhexosamine dimers [10–12].

### Calibration and sample preparations

Calibrators including CS, DS, and HS were purchased from Sigma Co. (Saint Louis, MO, USA). The stock solution of individual standard was prepared in a concentration of 1 mg/mL. The calibration curve was made by adding five different volumes for each GAG standard, and the final concentrations of five mixed working standards were 12.5, 25, 50, 100, and 200 μg/mL for DS; 6.25, 12.5, 25, 50, and 100 μg/mL for CS; 3.125, 6.25, 12.5, 25, and 50 μg/mL for HS, respectively. The five prepared GAG working standard mixtures (70 μL) and the precipitated GAG sample (2 μL) were added into tubes separately, and then, all the tubes were dried by nitrogen gas using the following methanolysis processes. The methanolysis was performed by adding 3 N HCl in methanol (200 μL) and 2, 2-dimethoxypropane (10 μL). After drying under nitrogen, acetonitrite (100 μL) and a mixture of GAG internal standards (100 μL) were added, and dried again. Reconstitution of the standards and samples was performed with 100 μL ammonium acetate (NH_4_OAc; 10 mM). The reconstituted fluids were filtrated and centrifuged by using a 3 kDa Amicon filter in 12,000 rpm for 30 minutes. After this, the filtrates were ready for LC-MS/MS analysis.

### Internal standard preparations

The internal standards including [^2^H_6_] DS, [^2^H_6_] CS, and [^2^H_6_] HS, were derived in-house from deuteriomethanolysis of GAG standards comprising DS, CS, and HS, according to the method reported by Zhang et al. [20]. The formation of ^2^HCl in C^2^H_3_O^2^H (2 mol/L) was first performed by adding a drop of acetyl chloride (CH_3_COCl) into deutrioacetate (C^2^H_3_O^2^H) on ice. The stock solution of individual GAG standard (300 μL) was added into a tube separately, and dried by nitrogen for 15 minutes. After drying, ^2^HCl in C^2^H_3_O^2^H (300 μL) was added and incubated in 65°C for 75 minutes. After being dried by nitrogen for about five minutes, deionized water (2 mL) was added and stored in ice (−20°C). A mixture of GAG internal standards with the same volumes of [^2^H_6_] DS, [^2^H_6_] CS, and [^2^H_6_] HS was ready for LC-MS/MS analysis. The GAG internal standard mixture was stable in 4°C for seven days.

### Experimental parameters of LC-MS/MS analysis

The LC-MS/MS analysis was performed on a 4000 QTRAP triple-quadrupole MS system equipped with a TurboIonSpray, and Agilent 1260 Infinity HPLC pump and autosampler. The 3-μm Atlantis dC18 column (3.0 × 50 mm) was purchased from Waters Corporation (Milford, MA, USA). The mobile phase A consisted of 5 mM NH_4_OAc (pH 5.5) in deionized water containing 0.1% formic acid and the mobile phase B consisted of 5 mM NH_4_OAc (pH 5.5) in 100% methanol, delivered in a gradient elution starting at 100% water and ending at 100% water at a flow rate of 450 μL/min. The injection volume was 5 μL, and the total run time was 6.5 minutes. The instrument was operated in the positive-ion mode. The experimental parameters were set as followed: curtain gas at 10 psi, CAD set at medium, IonSpray voltage at 5500 V, temperature at 450°C, Gas 1 at 50 psi, and Gas 2 at 60 psi. Nitrogen was used for the curtain gas, Gas 1, Gas 2, and collision gas. Data were acquired and processed using Analyst 1.5.2™ software (AB Sciex).

The calibration curve was made by plotting the standards with six concentrations including 0 μg/mL (X-axis) and their integrated areas (Y-axis) after MS/MS measurements. The linear range was set from 12.5 to 200 μg/mL for DS; 6.25 to 100 μg/mL for CS; and 3.125 to 50 μg/mL for HS. The “linear through zero” regression was excellent (y = 9.35e + 0.004×; *r* = 0.9994). The within-run and between-run precisions of the assay were determined by assay of three samples in triplicate over a period of three days. In this study, the triplicate results in low (10 μg/mL for DS; 2.5 μg/mL for HS), medium (50 μg/mL for DS; 20 μg/mL for HS), and high levels (150 μg/mL for DS; 40 μg/mL for HS) from three runs were used to estimate within-run and between-run precisions.

## Results

### Transition mass-to-charge ratio (*m/z*) of GAG standards and internal standards

One particular disaccharide for each GAG was selected, in which the parent ion and its daughter ion after collision were *m/z* 426.1 → 236.2 for DS (retention time = 3.30 ± 0.06 minutes) and CS (retention time = 3.75 ± 0.11 minutes), and *m/z* 384.2 → 161.9 for HS (retention time = 3.43 ± 0.09 minutes). The mass spectrum of the deuteriomethanolysis product of DS showed the incorporation of +6 atoms of deuterium, the same as that of HS. The protonated molecular ion was the transition *m*/*z* 432 → 239 for [^2^H_6_] CS and [^2^H_6_] DS dimers (retention time = 3.71 ± 0.09 minutes and 3.26 ± 0.05 minutes, respectively), as well as the molecular ion transition *m*/*z* 390.2 → 168 for the [^2^H_6_] HS dimer (retention time = 3.39 ± 0.06 minutes). Quantifications were achieved using peak areas that were processed using Analyst 1.5.2™ software (AB Sciex).

### Precision and recovery analyses of the tandem mass spectrometry assay

The within-run and between-run precisions were determined by an assay of total 18 samples in low, medium, and high concentrations of DS and HS using the method described earlier. All 18 samples were run in triplicate for within-run analysis, and a duplicate analysis of each sample (n = 18) was performed on three different days sequentially. The average within-run and between-run CV was 9.7% (low conc.), 6.4% (medium conc.), and 5.9% (high conc.), as well as 11.8%, 7.6%, and 9.3%, respectively. The analytical recovery was also investigated by spiked standards with known concentrations of DS and HS (20 μg/mL for each standard) into pooled normal urine samples as in the precision studies. The recoveries of this LC-MS/MS assay were 94.3% for DS (ranging from 91.9% to 97.6%), and 95.1% for HS (ranging from 92.3% to 98.4%). The linearity study of this method was carried out with concentrations ranging from 12.5 to 200 μg/mL for DS, and 3.125 to 50 μg/mL for HS. Each concentration was performed in triplicate. Linearity of DS and HS was calculated individually and the correlation coefficients (r) were 0.9914 for DS and 0.9935 for HS, respectively.

### Overall distributions of DS and HS in MPS patients and normal controls

**Figure 1:**
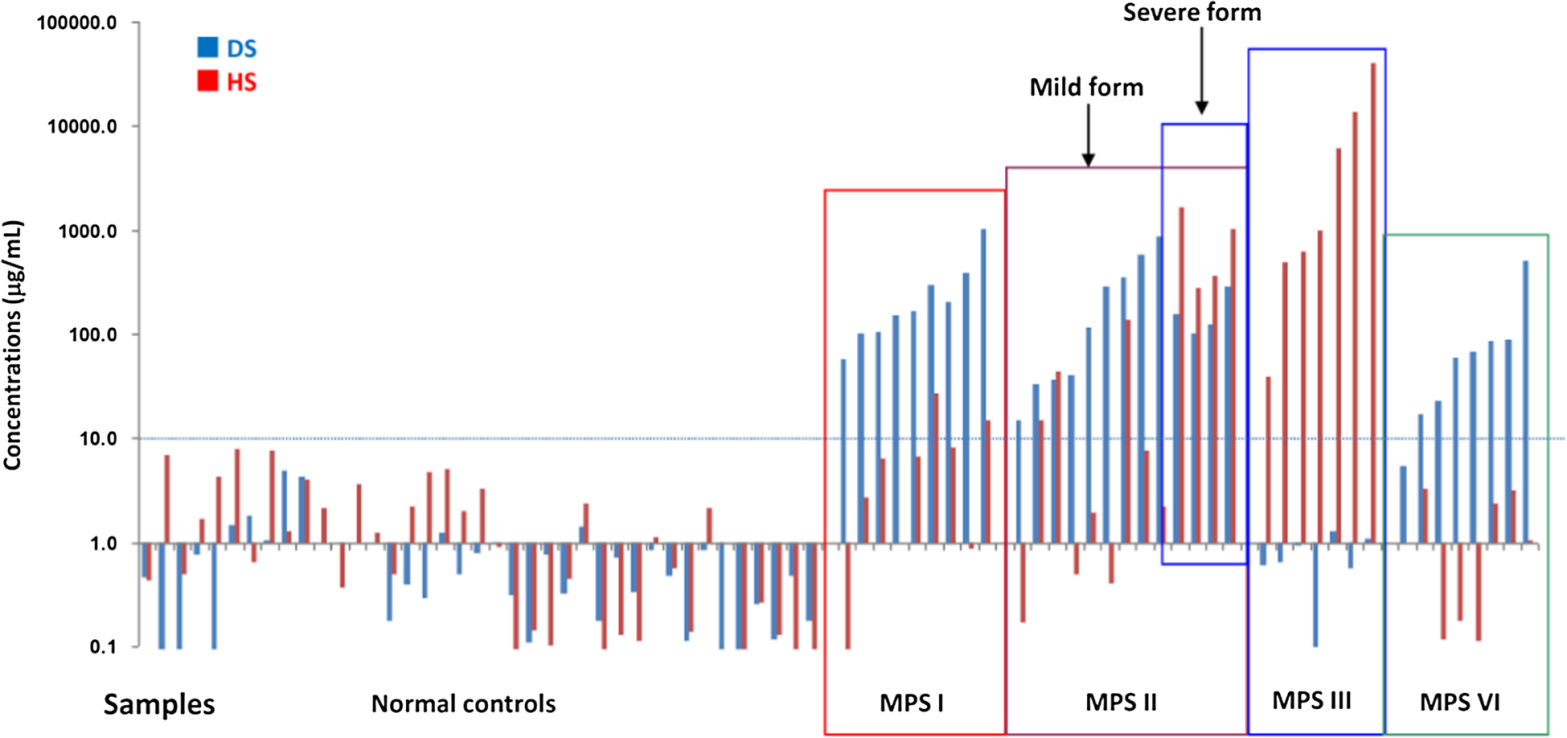
**The overall quantitations of DS and HS in urine of different MPS types and normal controls (n = 9 for MPS I, 13 for MPS II, 7 for MPS III, 8 for MPS VI, and 39 for normal controls).**

In this study, a total of 76 urine samples were collected and analyzed. As shown in Figure 1, normal samples could be easily distinguished from MPS patients based on the cut-off point (about 10 μg/mL) determined from overall urinary DS and HS measurements. In the control group, the only GAG that could be identified in urine was CS; there was no or very little DS and HS. In the MPS I and II patients, there was primarily an accumulation of both DS and HS, resulting in enhanced excretion in urine. In the MPS III patients, the affected GAG was HS, and in the MPS VI patients, the affected GAG was primarily DS.

### The mass spectrum of different MPS types and normal controls

**Figure 2:**
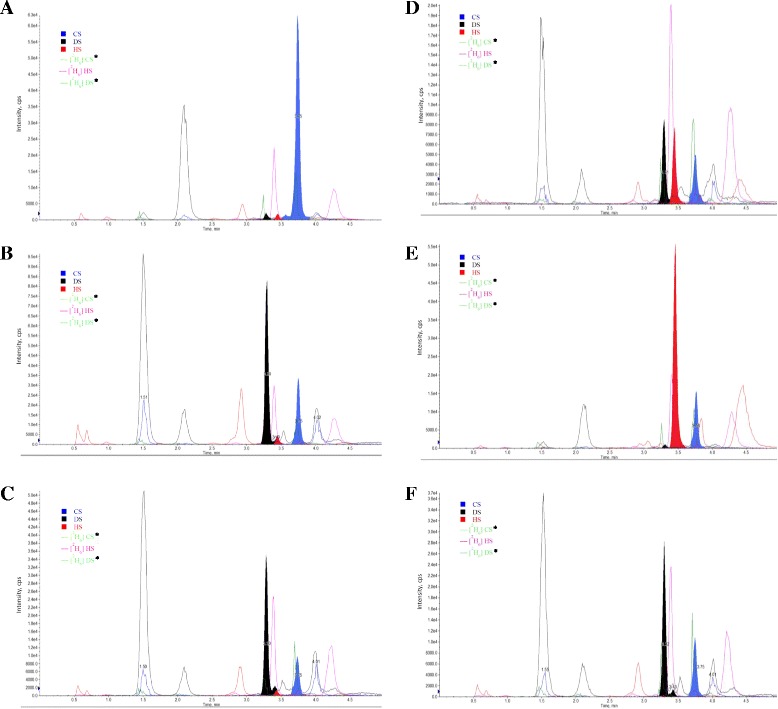
**Mass spectrum of DS and HS in urine, as well as the internal standards of different MPS phenotypes and normal controls. (A)** Normal control; **(B)** MPS I- Scheie; **(C)** mild form of MPS II; **(D)** severe form of MPS II; **(E)** MPS IIIB; and **(F)** MPS VI. *Same pairs of transition mass-to-charge ratio for [^2^H_6_] CS and [^2^H_6_] DS dimers (*m*/*z* 432 → 239), and the retention time (minutes) for [^2^H_6_] CS and [^2^H_6_] DS dimers is 3.71 ± 0.09 and 3.26 ± 0.05 minutes, respectively.

The mass spectrums of the normal control and each MPS type are illustrated in Figure 2 (A-F). The major urinary GAG found in normal control was CS, and with very small amounts of DS and HS, but not significant (Figure 2A). The only predominant similarity of affected GAGs among MPS I, II, and VI patients was DS; HS was found particularly in MPS I and II patients. The results showed a significant elevation of DS excreted in the urine of MPS I, II, and VI patients; levels of HS varied among them. Elevated excretion of HS was observed most significantly in the severe form of MPS II, and then, in the mild forms of MPS II, MPS I and MPS VI patients in decreasing order of elevations. The quantity of urinary HS in MPS VI patients was very little amount, and almost equivalent to that of the normal value. For those with MPS III, HS was the principal GAG that was grossly accumulated and excreted in urine, and showed a distinct HS mass spectrum diagram (Figure 2B-F).

### The concentrations of urinary DS and HS in different MPS types and normal controls

**Figure 3:**
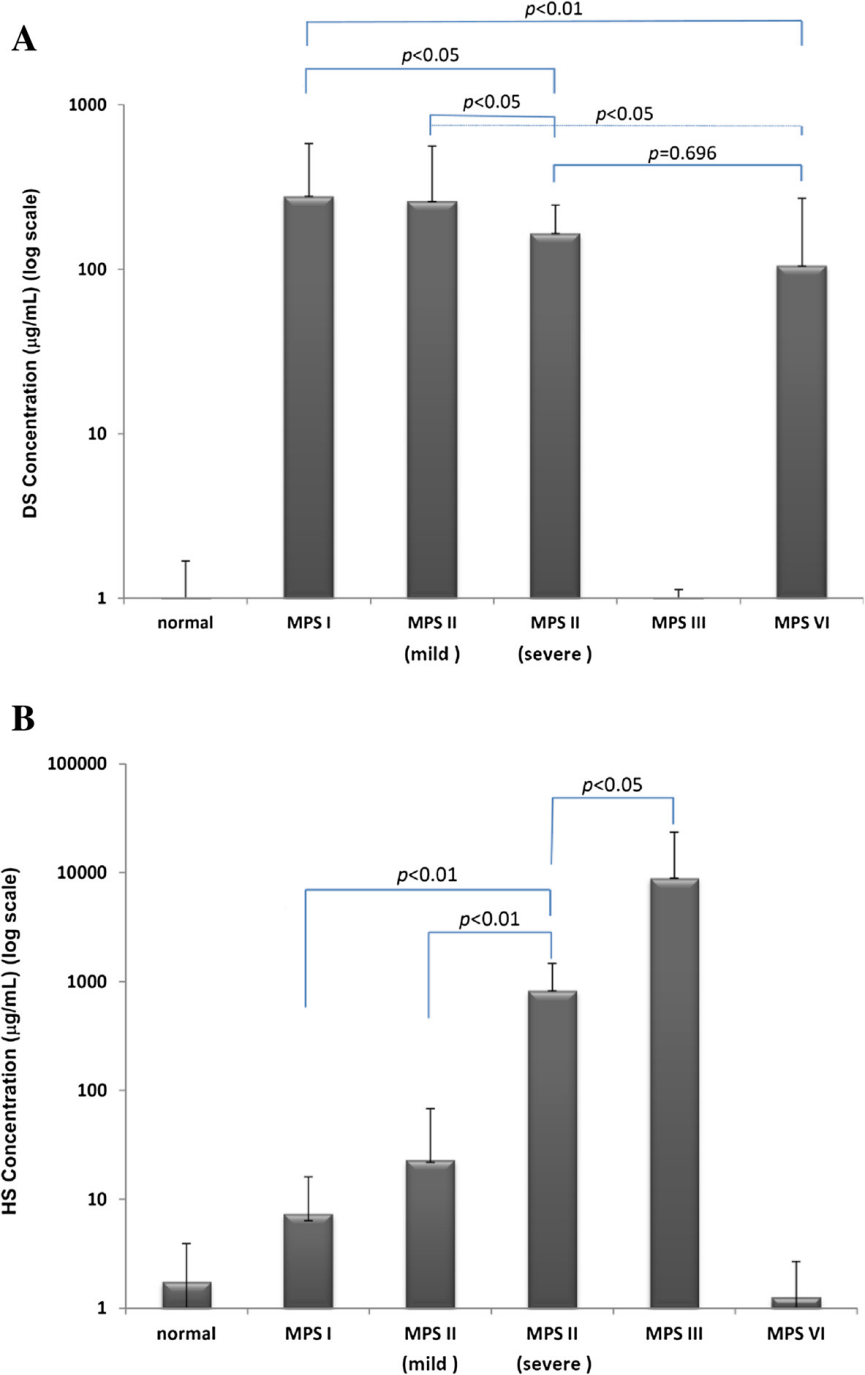
**The comparison of affected GAGs showed significant differences among different MPS types (n = 9 for MPS I; n = 5 for mild form of MPS II, and n = 8 for severe form of MPS II; n = 7 for MPS III; and n = 8 for MPS VI), and normal control (presented in log scale).** Urinary DS **(A)** and HS **(B)** concentrations in average (μg/mL) detected in different MPS types and normal controls.

The average concentrations (in μg/mL) of urinary DS and HS found in different MPS types and the normal controls were determined. The majority of urinary GAG found in normal control was CS (71.9 ± 46.6 μg/mL) with very little DS and HS (less than 1.68 ± 1.01 and 3.88 ± 2.16 μg/mL, respectively). In MPS I patients, DS and HS were simultaneously detected and were shown to be significantly elevated when comparing with normal control (*p* < 0.005 for DS; *p* < 0.001 for HS), and the concentrations were 276.9 ± 301.0 and 7.3 ± 8.7 μg/mL, respectively. When comparing the quantities of DS and HS in MPS I and II patients, the urinary HS in the severe form of MPS II was significantly greater than that of MPS I and the mild form of MPS II (824.0 *vs.* 7.3 and 22.9 μg/mL; *p* < 0.01), and the DS was slightly lower than that found in MPS I and the mild form of MPS II patients (164.6 *vs*. 276.9 and 258.6 μg/mL; *p* < 0.05) (Figures 3A and B). HS was predominant in the urine of MPS III patients, with an approximate average amount of 8901.4 μg/mL, which was thousands of times higher than the amounts seen in the normal control group. The differences were all significant when comparing within the different MPS types (*p* < 0.01). A very small amount of DS (0.73 μg/mL) was found in MPS III patients. The predominantly affected GAG in MPS VI patients was DS, which was seen to be moderately elevated, approximately 105.5 μg/mL on average, and a relatively little amount of HS (1.26 μg/mL) was observed. No significant difference was found when comparing DS values between the severe form of MPS II and MPS VI. This was a reliable indicator to discriminate MPS VI from MPS I and II (*p* < 0.01 and *p* < 0.05). The quantifications of DS and HS in different MPS types are compared and shown in Figures 3A and B (log scale).

### Evaluation of the effectiveness of enzyme replacement therapy (ERT) in MPS patients

**Figure 4:**
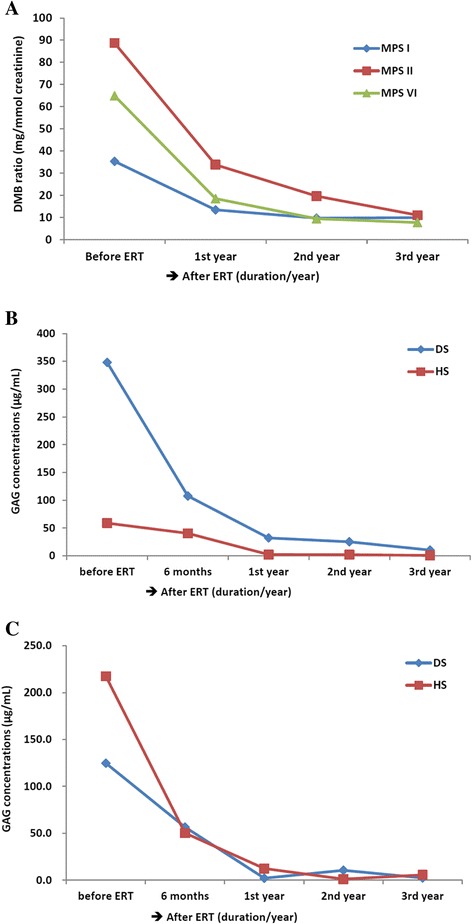
**Evaluations of the effectiveness of ERT in MPS patients.** The reduction of urinary GAGs after ERT in MPS I, II, and VI patients was significant **(A)**; the reduction of DS and HS in patients with MPS I- Scheie **(B)**, and the mild form of MPS II **(C)** patients after ERT was notable.

To evaluate the amelioration of MPS patients receiving ERT, the reduction of urinary GAG (DMB/creatinine ratio) and particular disaccharide units of the affected GAGs were measured for three successive years. The average reductions of urinary GAGs after ERT in MPS I (n = 9), II (n = 13), and VI (n = 9) patients were significant. In the first year, the reductions were 62.0%, 71.5%, and 61.9%, respectively. In the second year, they were 72.5%, 85.5% and 77.9%. In the third year, they were 71.9%, 88.1% and 87.6% (See Figure 4A). For MPS I-S and the mild form of MPS II patients, the reductions of DS and HS were also prominent. Respectively, they were 69.1% and 54.7%, as well as 31.5% and 76.9% in the first six months; 90.8% and 98.4%, as well as 96.4% and 94.3% in the first year; and a greater than 97.1% reduction for both in the second and the third years (Figures 4B and C).

## Discussion

The aim of this research project is to develop a LC-MS/MS method for MPS type determination instead of the conventional 2-D EP method. The data showed that the LC-MS/MS method was highly specific and highly sensitive to discriminate well among DS, HS, and CS, even with a trace of GAG content in the urine sample. It is encouraging to see that the results of this study are successful and can be used to distinguish one MPS type from the others.

Two main merits of our method that differ from the method reported by Auray-Blais et al. are sample itself and the addition of internal standards. According to the method previously reported by Auray-Blais et al., a small amount of the urine sample is directly methanolyzed without undertaking GAG precipitation. By doing it this way, a black sticky analyte is formed, which cannot be successfully introduced into LC-MS/MS for further analysis as it obstructs the HPLC flow and column. By performing the GAG precipitation, the feasibility of CS, DS, or HS detections in the urine sample could be significantly enhanced. Without a doubt, the addition of stable-radioisotope internal standards (IS) is another merit of our method modification. Combined with the addition of internal standards, the eluted positions of CS, DS, or HS can be precisely identified. In addition, the relative concentrations of CS, DS, and HS in the urine sample can be obtained by integrating the peak areas between the individual GAG and that of internal standard with known concentration. The internal standard can also be used as a quality assurance in LC-MS/MS analysis.

Based on the results detected by LC-MS/MS method, the quantities of affected GAGs are distinctive for MPS type determinations. The results are fully matched with those examined by 2-D EP method, and correspond well with the leukocyte enzymatic assay. The LC-MS/MS method used for MPS type determination has many advantages, including high sensitivity, high specificity, high throughput, high reliability, and analytic automation. The highly sensitive of LC-MS/MS method was achieved and the lower limit of quantitation (LLOQ) was less than 0.06 μg and 0.065 μg with 100 μL urine for DS and HS, respectively. The high specificity of MRM detection allowed specific detection of DS and HS, in which the parent ion and its daughter ion after collision were *m/z* 426.1 → 236.2 for DS and *m/z* 384.2 → 161.9 for HS.

The LC-MS/MS method can also be used for MPS subgroup classifications, particularly for MPS II. In this application, the concentration of HS is the key factor used to distinguish the mild form of MPS II from the severe form. The results obtained in this study showed that the concentrations of HS detected from the samples of the severe form MPS II patients were significantly higher than those of the mild form MPS II patients. The increase was over 35 times greater (824.0 *vs*. 22.9 μg/mL). The results also showed that the concentrations of DS were varied among MPS I, MPS II, and MPS VI. The quantities of DS detected from MPS VI patients was significantly lower than those with MPS I and the mild form MPS II patients; but was equivalent to those of severe form MPS II patients. As very little or no HS was seen in the samples from MPS VI patients, and this can be used as a marker.

Disease-specific treatments to replace specific enzyme activity include ERT for the non-neurologic manifestations of MPS patients [22–31], and bone marrow as well as hematopoietic stem cell transplantations for MPS I, II, IV, and VI patients [32,33]. de Ru et al. reported that ERT can result in good clearance of GAGs from many tissues and can significantly ameliorate several symptoms [29]. The effectiveness of ERT for patients with MPS I, II, and VI is significant. In general, the improvement of symptoms or physiological activities is one major indicator used to evaluate the effectiveness of ERT, as is, the clearance of GAGs from urine. Notably, the DMB ratio significantly ameliorated and declined to 55% to 65% of the original value when MPS patients had received ERT for more than three to six months. The DMB ratio can give useful information to evaluate the effectiveness of ERT; however, it cannot show which GAG can be degraded well after ERT. HS is mainly found in the central nervous system and it accumulates in the brain if it cannot be broken down due deficient or missing enzymes. By using the LC-MS/MS method, this study showed that the concentrations of both DS and HS are reduced significantly when comparing the predominant disaccharide units in MPS I and II patients before and after ERT. The results demonstrate that the reductions of DS and HS after ERT are substantial, and the value of predominate disaccharide unit detections by using LC-MS/MS method is suitable for routine purposes and useful in evaluating the MPS progressive conditions.

## Conclusions

The LC-MS/MS method used for MPS type determination is specific, sensitive, validated, reproducible, accurate and applicable for simultaneous quantification of urinary DS and HS. By using this method, MPS can be properly diagnosed. As well, this method can be used to evaluate the effectiveness of follow-up therapy.
